# Long-term genetic selection reduced prevalence of hip and elbow dysplasia in 60 dog breeds

**DOI:** 10.1371/journal.pone.0172918

**Published:** 2017-02-24

**Authors:** A. M. Oberbauer, G. G. Keller, T. R. Famula

**Affiliations:** 1 Department of Animal Science, University of California, Davis, Davis, CA United States of America; 2 Orthopedic Foundation for Animals, Columbia, MO United States of America; University of Illinois, UNITED STATES

## Abstract

Canine hip dysplasia (CHD) and elbow dysplasia (ED) impact the health and welfare of all dogs. The first formally organized assessment scheme to improve canine health centered on reducing the prevalence of these orthopedic disorders. Phenotypic screening of joint conformation remains the currently available strategy for breeders to make selection decisions. The present study evaluated the efficacy of employing phenotypic selection on breed improvement of hips and elbows using the Orthopedic Foundation for Animals complete database spanning the 1970–2015 time period. Sixty breeds having more than 1000 unique hip evaluations and 500 elbow evaluations (1,056,852 and 275,129 hip and elbow records, respectively) were interrogated to derive phenotypic improvement, sex and age at time of assessment effects, correlation between the two joints, heritability estimates, estimated breeding values (EBV), and effectiveness of maternal/paternal selection. The data demonstrated that there has been overall improvement in hip and elbow conformation with a reduction in EBV for disease liability, although the breeds differed in the magnitude of the response to selection. Heritabilities also differed substantially across the breeds as did the correlation of the joints; in the absence of a universal association of these differences with breed size, popularity, or participation in screening, it appears that the breeds themselves vary in genetic control. There was subtle, though again breed specific, impact of sex and older ages on CHD and ED. There was greater paternal impact on a reduction of CHD. In the absence of direct genetic tests for either of these two diseases, phenotypic selection has proven to be effective. Furthermore, the data underscore that selection schemes must be breed specific and that it is likely the genetic profiles will be unique across the breeds for these two conditions. Despite the advances achieved with phenotypic selection, incorporation of EBVs into selection schemes should accelerate advances in hip and elbow improvement.

## Introduction

Canine hip dysplasia (CHD) is a highly prevalent inherited condition affecting dogs [1–3] irrespective of whether the dog is purebred or not [4, 5] although some breeds have a reported higher incidence than others [6]. Although normal at birth [7], the multifactorial nature of hip joint development including both genetic and environmental contributions makes it susceptible to laxity of the coxo-femoral joint, subluxation, and alteration of the femoral head and acetabulum. Hip joint laxity permits abnormal joint wear and ultimately the development of irreversible osteoarthritis, bone spurs, and degenerative joint disease [8, 9]. The condition ranges from mild to debilitating and severe [10]. There are several international schemes for assessing CHD some incorporating objective measurements of the ventral dorsal hip-extended radiograph whereas others utilize a subjective assessment of radiographic features [11].

For decades, there have been concerted efforts to characterize and reduce the incidence CHD [12, 13]. Reports in the literature indicate variable response to genetic selection based upon phenotypic radiographic evidence of CHD in individual dogs (recently reviewed by [14]. Breeding schemes reliant upon phenotype have shown a modest [15, 16] or more substantial [1, 17–19] degree of improvement in hip conformation and reduced incidence CHD. Some of the variability across findings may represent differences in sample size, hip assessment protocols, preferential sire selection, and breed contribution in the study design; the latter is especially important as larger breeds are typically more prone to express these conditions due to either genetic ancestry [20] or conformational morphology [21]. A complicating factor in reducing CHD is the variability of heritability estimates that range from 0.11 to 0.68 [22] that probably reflects breed differences [14], as well as the influence of environmental factors and whether the assessment scheme is voluntary wherein owners may elect to only submit dogs with acceptable joint morphology.

Elbow dysplasia (ED) is another orthopedic condition long recognized in the dog [23] with certain breeds having higher prevalence [9, 24, 25]. Presentation of the condition can be as early as 6 months or much later as a consequence of continued joint deterioration [26]. Elbow dysplasia, as defined by the International Elbow Working Group [27], represents a composite of traits that include ununited anconeal process, fragmented medial coronoid process, osteochondrosis or osteochondritis dissecans, and incongruity of the elbow joint [28]. Together these developmental anomalies comprise a diagnosis of ED and can be associated with pain, forelimb lameness, and reluctance to extend or flex the elbow joint. Common diagnosis of ED is through radiographic evaluation of the elbow joint [29].

Retrospective analyses of epidemiological data have revealed a genetic basis to ED with heritability estimates of 0.10 to 0.77 and males showing a greater heritability than females [30–32]. Similar to CHD, the high variability in estimates likely reflects breed differences coupled with differential heritabilities associated with the four traits that comprise the umbrella ED diagnosis [33] as well as the voluntary nature of some evaluation schemes.

The risk of concomitant expression of CHD and ED has been estimated to be 1.67 with an increased correlated risk when either joint has severe dysplasia [34] [35]. The studies that have assessed genetic correlation between CHD and ED have found minimal correlation across breeds with modest correlations seen for some breeds [1, 32, 36, 37]. The low correlations identified in the various studies suggest that although some bone formation genes may be common to both joints, the majority of genetic regulatory elements are distinct. It is also probable that the known environmental contributions which influence expression of both CHD and ED, such as nutrition, growth rate, and overall body weight, may impact the two disorders differentially. Selection to reduce prevalence of one condition may or may not result in improvement in the other [1, 15].

The objectives of the present study were to characterize the influence of non-compulsory selection on phenotypic radiographic assessment of hip and elbow conformation over time. Dog breeds that were most highly participatory in a voluntary United States radiographic screening process for hips and elbows were evaluated for improvement, whether maternal or paternal selection was more responsible for any observed progress, whether some breeds prove more amenable to selection than others, and whether selection against one orthopedic disorder yielded concomitant improvement in the other.

## Materials and methods

Data: The complete Orthopedic Foundation for Animals (OFA) database for hip and elbow ratings on dog breeds with more than 1000 records for hips and 500 records for elbows was interrogated. Data evaluated included those publically released and those withheld from public release from 1970 through April 2015. The OFA categorizes hips as excellent, good, fair, borderline, mild dysplasia, moderate dysplasia, severe dysplasia with a single rating that encompasses both hip configurations. For the analyses, hip scores were classified as follows: 1 as excellent, 2 as good, 3 as fair, 4 as borderline, 5 as mild, 6 as moderate, and 7 as severe. Hip scores 1–3 are considered non-dysplastic. Elbow scores were classified similarly with 1 as normal, 2 as degenerative joint disease grade I, 3 as degenerative joint disease grade II, and 4 as degenerative joint disease grade III.

### Statistical analyses

Hip and elbow scores are ordered categories, suggesting a threshold model with a probit link function [38] as a natural procedure for any analysis. Accordingly, we consider an algebraic form for the risk score in either disease measure, within a breed, to take the form:

$${\text{y}}_{\text{ijknm}}=\mu_{\text{m}}+{\text{sex}}_{\text{jm}}+\beta_{\text{m}}{\left(\text{age at diagnosis}\right)}_{\text{j}}+{\text{year of diagnosis}}_{\text{km}}+{\text{animal}}_{\text{ijknm}}+{\text{e}}_{\text{ijknm}}$$ (1)

where y_ijknm_ is the underlying risk variable of the m-th trait (m = hip or elbow) on the n-th dog (n = 1,2,…n_breed_) of the i-th sex (i = M or F), diagnosed in the k-th year (k = 1970, 1971, …2012) and diagnosed at the j-th age in months (j = 28, 29, … 841). μ_m_ is a constant common to all dogs for a given joint trait and sex_im_ is the effect of sex i on trait m. β_m_ is a regression coefficient relating the m-th trait score to the age of the dog at diagnosis. Following that are terms for the k-th year of diagnosis effect of the m-th trait and the genetic contribution each animal brings to its own hip or elbow score (i.e., animal_ijknm_). The year of evaluation was computed from the data file using date of birth and age at diagnosis. Finally, e_ijknm_ represents the remaining residual, or any unexplained variability in hip and elbow scores.

The animal contributions for a given trait, animal_ijknm_, can be represented as a vector of breeding values for all dogs identified in the data set of a given breed. This can be represented as two vectors of equal length (that length being the number of animals in the breed pedigree), **a**_h_ and **a**_e_, for the traits of hips and elbows, respectively. Accordingly, we assume

$$\text{var}\left[\begin{array}{c} {\text{a}}_{\text{h}}\\ {\text{a}}_{\text{e}} \end{array}\right]={\text{G}}_{0}⨂\text{A}=\left[\begin{array}{cc} {\text{g}}_{\text{h}} & {\text{g}}_{\text{he}}\\ {\text{g}}_{\text{he}} & {\text{g}}_{\text{e}} \end{array}\right]⨂\text{A}$$

with **A** being the numerator relationship matrix [38], **G**_0_ containing the genetic variances for hips and elbows (g_h_ and g_e_) along with the genetic covariance between hips and elbows (g_he_) and the symbol ⊗ representing a direct product of the two matrices **G**_0_ and **A**.

In a similar fashion, the residuals for each trait, e_ijknm_, can also be represented as two vectors **e**_h_ and **e**_e_ for hips and elbows, respectively, random variables assumed to be uncorrelated with the genetic effects in **a**_h_ and **a**_e_. The variances for each residual term (call them r_h_ and r_e_) are set to 1.0, a necessary component for the fitting of mixed categorical models. However, for those individuals with observations on both traits we can consider the parameter r_he_, the covariance between residuals for the risk of hip and elbow disease. The covariance structure of residuals for this subset of animals that have a recorded hips and elbow score would then take the form

$$\text{var}\left[\begin{array}{c} {\text{e}}_{\text{h}}\\ {\text{e}}_{\text{e}} \end{array}\right]={\text{R}}_{0}⨂\text{I}=\left[\begin{array}{cc} {\text{r}}_{\text{h}} & {\text{r}}_{\text{he}}\\ {\text{r}}_{\text{he}} & {\text{r}}_{\text{e}} \end{array}\right]⨂\text{I}=\left[\begin{array}{cc} 1 & {\text{r}}_{\text{he}}\\ {\text{r}}_{\text{he}} & 1 \end{array}\right]⨂\text{I}$$

where **R**_0_ is a matrix of residual variances (r_h_, r_e_) and covariance (r_he_) for hips and elbows, I is an identity matrix of order the number of dogs scored for both hips and elbow disease and, as above, ⊗ signifies a direct product.

The objective was to estimate the unknown fixed effects for each trait (μ_m_, sex_im_, β_m_ and year of diagnosis_km_) and to predict the random effects (animal_ijknm_), along with estimation of the unknown genetic variances (g_h_, g_e_), the genetic covariance (g_he_) as well as the residual covariance (r_he_). In addition, we can estimate the heritability of risk for hip disease as h^2^_h_ = g_h_ / (g_h_ + 1) and that for elbow disease as h^2^_e_ = g_e_ / (g_e_ + 1), along with the genetic correlation between these traits as r_g_ = g_he_ /√(g_h_ g_e_). Finally, we can also use the predicted breeding values to estimate the impact of selection in each of these traits across the 60 breeds represented in this data set.

A Bayesian framework, a strategy of considerable power and plasticity, was used to evaluate these unknown values [39]. The public domain package MCMCglmm [40], available through the language R [41], was used. The prior distributions for the putative fixed effects (i.e., the non-genetic effects of μ_m_, sex_im_, β_m_ and year of diagnosis_km_) were independent normal densities with null means and variances of e^10^, that is, a diffuse normal prior. The prior densities for the putative random effects (i.e., the animal effects, animal_ijknm_) were multivariate normal densities with null means and covariance represented in G_0_ (g_h_, g_e_ and g_he_. The prior distribution for the variance and covariance components (σ^2^_ah_, σ^2^_ae_ and σ_he_) was assumed to be an inverse-Wishart density. For variance components the inverse-Wishart is governed by two parameters, V and nu in the notation of [40]. The distribution is right-skewed when nu is small, with a mode of V*nu/(nu+2). For our analyses we set nu = 0.002 to permit for a long flat right-skewness, effectively creating a diffuse prior for the unknown variances. The MCMCglmm package [40] refers to nu as the “degree of belief parameter”, the smaller the value, the weaker one’s prior belief in the value V. The unknown residual covariance, r_he_, was also assumed to follow an inverse-Wishart density, necessarily constrained to ensure that the covariance matrix **R**_0_ was positive definite [40].

As implied by the use of the MCMCglmm package, estimates of the posterior density for the unknown parameters were generated through a Monte Carlo Markov chain. Each trait was evaluated in one continuous chain, each starting at disperse values for the unknown variances. Convergence was examined through trace plots making use of the R package coda [42]. Each chain was run a total of 100,000 rounds with a burn-in of 20,000 rounds and a thinning interval of 20 (creating a single chain sample of 4,000 values). The model parameterization was considered valid and the model converged if the autocorrelation between successive stored iterations was less than 0.1, the time series of the parameters for the MCMC iterates show no obvious trends, and the posterior density estimates of the parameters meet the assumption of normality [40].

In the process of estimating heritability, we are also able to extract the additive genetic merit of all dogs in each pedigree. These estimated breeding values (EBVs) can be used to evaluate the response to selection on hip and elbow disease by participating breeders. The estimation of this genetic trend, done on several of the 60 breeds represented in the data set, is no more than the arithmetic mean of the EBVs of all dogs in each year of birth. Plotting these values, though on the unobserved probit scale, does permit an assessment of the changing genetic profile of the breed over time. Data were then fitted to a loess regression within the ggplot2 package [43] in the public domain package R [41].

In a few selected instances, we also chose to deconstruct the observed selection response across the four paths of possible genetic improvement that are often used in livestock improvement. That is, to source the response to selection through the differing intensities and accuracies typically occurring in the selection of sires of sires (SS), sires of dams (SD), dams of sires (DS) and dams of dams (DD). This technique was first proposed many years ago in dairy cattle [44]. This calculation requires that one first identifies those animals that produce grand-progeny in the pedigree. In this instance, we calculated the simple arithmetic mean of the EBVs for the animals, both males and females, that fell into one of the four grand-parental categories (i.e., SS, SD, DS or DD) producing values independent of the number of grand-offspring produced. This simple, unweighted, calculation provides an examination of the history of decision making within the selected breed, identifying animals that should have had some impact on the trajectory of genetic improvement seen in the population as a whole. Of course the number of animals in each of these groups is much smaller than that of the whole population when grouped by year of birth. Therefore, to simplify the assessment, the plot of the mean of each grand-parental group by year of birth was evaluated by fitting a quadratic polynomial regression (with year of birth as the independent variable) within the ggplot2 package [43] in the public domain package R [41].

## Results

Restricting the OFA data to dogs 2 years of age and older at time of evaluation and to breeds with the requisite number of hip and elbow evaluations resulted in 1,056,852 and 275,129 hip and elbow records, respectively, representing 60 different breeds and in some cases more than eleven generations. The number of records that met the criteria for analyses varied by breed (S1 Table) with a high of 234,382 and 72,126 hips and elbow records, respectively, for the Labrador retriever to a low of 989 and 513 hip and elbow records, respectively, for the Tibetan mastiff.

The fifteen breeds with the highest proportion with dysplastic hip and elbow ratings are presented in Table 1. Nine of the breeds having the highest proportion of CHD also had the highest proportion of dysplastic elbow ratings. These breeds with the highest prevalence do not share any obvious phenotypes or ancestral relationships. Breeds having CHD proportions of less than 4% of the evaluated population were the German shorthaired pointer, the flat coated retriever, the Belgian tervuren, and the Belgian sheepdog (Table 2). Breeds with less than 1% of the evaluated population having ED were Doberman pinscher, German shorthaired pointer, Boxer, Flat coated retriever, Bichon frise, Cavalier King Charles spaniel, and Briard (Table 2).

**Table 1:** The 15 breeds with the highest prevalence of hip (CHD) and elbow dysplasia (ED), all dysplastic categories combined.

	% CHD		% ED
Newfoundland	24.8	Chowchow	48.6
Bloodhound	24.7	Rottweiler	38.1
American Staffordshire terrier	24.4	Bernese mountain dog	26.0
Bullmastiff	24.0	Chinese shar-pei	24.0
Rottweiler	20.1	Newfoundland	22.7
Chesapeake bay retriever	19.4	German Shepherd	17.8
Chowchow	19.2	American Staffordshire terrier	16.1
German Shepherd	18.9	Irish water spaniel	15.6
Golden	18.8	English setter	14.2
Mastiff	18.6	Bullmastiff	14.2
Gordon setter	18.3	Tibetan mastiff	13.8
Old English sheepdog	17.8	Mastiff	13.1
Giant schnauzer	17.7	Bloodhound	13.1
Pembroke Welsh corgi	16.6	Gordon setter	12.6
Greater Swiss mountain dog	16.6	English Springer	12.5

**Table 2:** The 15 breeds with the lowest prevalence of hip (CHD) and elbow dysplasia (ED), all dysplastic categories combined.

	% CHD		% ED
Belgian sheepdog	2.8	Briard	0.2
Belgian tervuren	3.1	Cavalier King Charles spaniel	0.3
Flat coated retriever	3.7	Bichon	0.5
German shorthaired pointer	3.9	Flat coated retriever	0.7
Shetland sheepdog	4.2	Boxer	0.7
Rhodesian Ridgeback	4.4	German shorthaired pointer	0.8
Irish wolfhound	4.5	Doberman pinscher	0.8
Belgian Malinois	5.2	Akita	1.1
Bearded Collie	5.3	Border collie	1.1
Australian shepherd	5.3	Portugese water dog	1.4
Doberman pinscher	5.7	Welsh springer spaniel	1.4
Keeshond	5.8	Great Pyrenees	1.5
Nova Scotia Duck Tolling Retriever	5.8	Weimaraner	1.6
Bichon	5.9	Brittany	1.7
Viszla	6.2	Samoyed	1.9

When evaluating a sex effect on hip ratings (S2 Table), in 14 breeds males had hip evaluations that were more favorable than females (p < 0.05) indicated with a negative value. In another 15 breeds, females had more favorable ratings than males indicated with a value that is positive and significantly different from zero; the remaining breeds had no significant sex effect. Males of 18 breeds had less favorable elbow ratings than females with the sex effect most pronounced in the Alaskan malamute. The Rhodesian ridgeback was the only breed in which males had a more favorable ED rating when compared to females.

Increasing age had a subtle but significant (p < 0.05) effect in 34 breeds with older dogs having, on average slightly higher ratings for hips (S2 Table). Age had a minor impact on elbow ratings in 16 breeds with three of those breeds, the Bichon, Chinese shar-pei, and Akita, having more favorable elbow ratings with increasing age.

Heritabilities for both joints differed by breed (Table 3) with an average overall 60 breeds of 0.57 and 0.29 for CHD and ED, respectively. The highest heritability for CHD was observed in the Boxer at 0.75 ± 0.02 and the lowest observed in the English setter at 0.46 ± 0.03. Fifty-two breeds had heritabilities in excess of 0.50 confirming opportunity for reasonable improvement with the application of classical selection pressure. Submission numbers were compared for the 15 breeds having the highest and lowest estimated heritabilities and they did not differ (p < 0.05). The Welsh springer spaniel had an estimated ED heritability of 0.90 ± 0.04 whereas the Cavalier King Charles spaniel had the lowest heritability at 0.01 ± 0.01. Only 11 breeds had ED heritabilities in excess of 0.50 and 13 breeds had estimated heritabilities below 0.1. The median number of submissions per breed for breeds having ED heritabilities > 0.5 or <0.1 did not differ (p < 0.05)

**Table 3:** Heritabilities for CHD and ED scores (mean + standard error of the mean) and the genetic correlation of the two joint scores for 60 dog breeds.

BREED	Heritability of Hip Score	Heritability of Elbow Score	Genetic Correlation of Hip and Elbow Score
Akita	0.47 ± 0.02	0.53 ± 0.10	0.34 ± 0.16
Alaskan malamute	0.62 ± 0.03	0.24 ± 0.04	0.99 ± 0.01
American Staffordshire terrier	0.54 ± 0.06	0.27 ± 0.13	0.75 ± 0.17
Anatolian	0.53 ± 0.07	0.17 ± 0.07	0.92 ± 0.09
Australian cattle dog	0.60 ± 0.03	0.39 ± 0.11	0.49 ± 0.16
Australian shepherd	0.56 ± 0.02	0.25 ± 0.08	0.81 ± 0.11
Bearded Collie	0.53 ± 0.04	0.08 ± 0.04	-0.68 ± 0.38
Belgian Malinois	0.68 ± 0.05	0.20 ± 0.08	0.57 ± 0.18
Belgian sheepdog	0.51 ± 0.04	0.15 ± 0.10	0.54 ± 0.27
Belgian tervuren	0.49 ± 0.04	0.16 ± 0.05	0.93 ± 0.08
Bernese mountain dog	0.47 ± 0.02	0.26 ± 0.02	0.51 ± 0.04
Bichon	0.55 ± 0.04	0.85 ± 0.04	0.92 ± 0.11
Bloodhound	0.54 ± 0.04	0.16 ± 0.06	0.94 ± 0.07
Border collie	0.61 ± 0.03	0.14 ± 0.11	0.53 ± 0.28
Bouvier	0.54 ± 0.03	0.34 ± 0.08	0.45 ± 0.14
Boxer	0.75 ± 0.02	0.23 ± 0.18	-0.36 ± 0.50
Briard	0.52 ± 0.07	0.20 ± 0.12	0.79 ± 0.43
Brittany	0.58 ± 0.02	0.16 ± 0.04	0.69 ± 0.21
Bullmastiff	0.52 ± 0.04	0.41 ± 0.06	0.51 ± 0.10
Cavalier King Charles spaniel	0.60 ± 0.03	0.01 ± 0.01	-0.58 ± 0.32
Chesapeake bay retriever	0.56 ± 0.02	0.04 ± 0.03	0.78 ± 0.24
Chinese shar-pei	0.59 ± 0.04	0.25 ± 0.09	0.47 ± 0.21
Chowchow	0.59 ± 0.03	0.19 ± 0.07	0.32 ± 0.18
Doberman pinscher	0.54 ± 0.02	0.02 ± 0.01	-0.82 ± 0.11
English setter	0.46 ± 0.03	0.46 ± 0.05	0.35 ± 0.107
English springer	0.65 ± 0.02	0.55 ± 0.05	0.44 ± 0.07
Flat coated retriever	0.49 ± 0.04	0.61 ± 0.10	0.60 ± 0.16
German shepherd	0.58 ± 0.03	0.26 ± 0.08	0.66 ± 0.13
German shorthaired pointer	0.53 ± 0.03	0.38 ± 0.14	0.18 ± 0.31
German wirehaired pointer	0.50 ± 0.05	0.05 ± 0.04	-0.68 ± 0.30
Giant schnauzer	0.57 ± 0.05	0.05 ± 0.03	0.32 ± 0.50
Golden retriever	0.65 ± 0.03	0.29 ± 0.11	0.50 ± 0.19
Gordon setter	0.58 ± 0.03	0.05 ± 0.02	0.91 ± 0.09
Great dane	0.55 ± 0.03	0.15 ± 0.03	0.98 ± 0.02
Great Pyrenees	0.56 ± 0.04	0.37 ± 0.17	0.57 ± 0.23
Greater Swiss mountain dog	0.57 ± 0.05	0.44 ± 0.07	0.38 ± 0.11
Havanese	0.64 ± 0.04	0.85 ± 0.03	0.07 ± 0.08
Irish setter	0.49 ± 0.03	0.86 ± 0.06	0.33 ± 0.14
Irish water spaniel	0.65 ± 0.06	0.16 ± 0.07	0.87 ± 0.13
Irish wolfhound	0.60 ± 0.09	0.52 ± 0.11	0.19 ± 0.17
Keeshond	0.57 ± 0.04	0.05 ± 0.03	0.90 ± 0.12
Labrador retriever	0.59 ± 0.05	0.10 ± 0.05	0.90 ± 0.11
Leonberger	0.69 ± 0.05	0.11 ± 0.05	0.91 ± 0.11
Mastiff	0.53 ± 0.03	0.32 ± 0.05	0.31 ± 0.09
Miniature American shepherd	0.49 ± 0.09	0.53 ± 0.11	0.96 ± 0.08
Newfoundland	0.51 ± 0.02	0.39 ± 0.03	0.35 ± 0.05
Nova Scotia duck tolling retriever	0.64 ± 0.05	0.25 ± 0.08	0.99 ± 0.01
Old English sheepdog	0.58 ± 0.03	0.08 ± 0.04	-0.24 ± 0.48
Pembroke Welsh corgi	0.54 ± 0.03	0.15 ± 0.05	0.96 ± 0.05
Poodle	0.60 ± 0.02	0.52 ± 0.09	0.17 ± 0.17
Portugese water dog	0.55 ± 0.03	0.06 ± 0.05	0.08 ± 0.45
Rhodesian Ridgeback	0.58 ± 0.03	0.07 ± 0.04	0.81 ± 0.13
Rottweiler	0.57 ± 0.02	0.68 ± 0.03	0.27 ± 0.03
Samoyed	0.52 ± 0.03	0.15 ± 0.10	0.39 ± 0.32
Shetland sheepdog	0.61 ± 0.02	0.28 ± 0.06	0.95 ± 0.06
Spinone Italiano	0.52 ± 0.11	0.04 ± 0.04	0.39 ± 0.50
Tibetan mastiff	0.71 ± 0.05	0.22 ± 0.12	0.82 ± 0.16
Viszla	0.65 ± 0.02	0.47 ± 0.10	0.47 ± 0.15
Weimaraner	0.52 ± 0.03	0.11 ± 0.07	0.16 ± 0.46
Welsh springer spaniel	0.59 ± 0.10	0.90 ± 0.04	-0.06 ± 0.23

The correlation between the two joint ratings also varied by breed with 14 breeds having correlations > 0.9 and the Alaskan malamute and Nova Scotia duck tolling retriever having nearly perfect correlation (0.99 ± 0.01 and 0.99 ± 0.01, respectively). In contrast, 19 breeds showed no significant correlation between the two joint ratings and the two joints were significantly negatively correlated in the Doberman pinscher.

An intensive examination of each of the 60 breeds represented in this study is beyond the scope of this publication. Therefore, we selected several breeds for closer inspection to examine the evidence for possible phenotypic and genetic progress in combatting joint disease. We examined several breeds of high prevalence, along with a comparison to several other breeds with a low prevalence of CHD. As presented in Table 1, the five breeds with the highest prevalence of hip disease are the Newfoundland, Bloodhound, American Staffordshire terrier, Bullmastiff and the Rottweiler. The five breeds with the lowest prevalence of CHD are the German shorthaired pointer, Flat coated retriever, Belgian tervuren, Belgian sheepdog, and the Shetland sheepdog.

**Fig 1:**
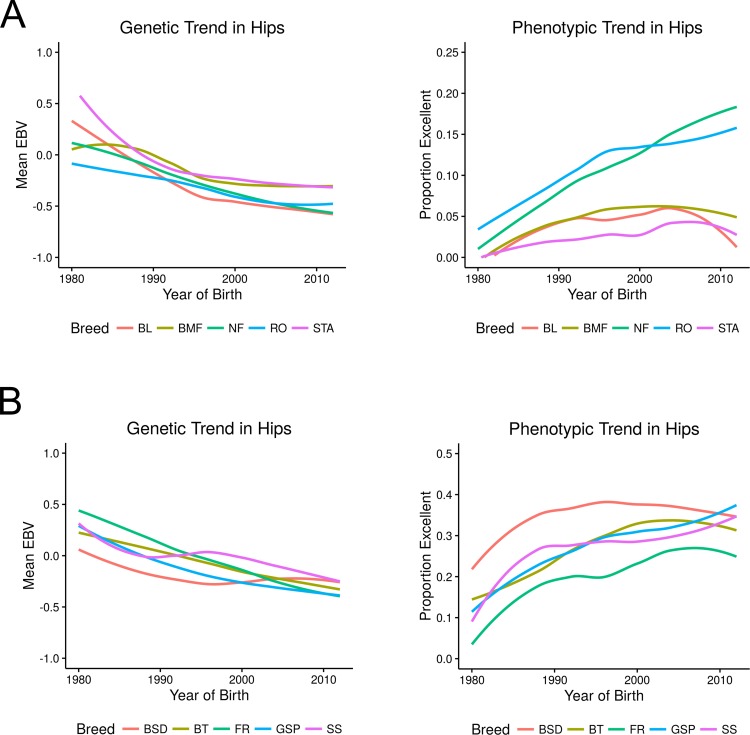
Genetic and phenotypic trends in CHD. (A) presents the genetic trend (EBV) and the phenotypic trend (proportion of hips rated as excellent) among the high prevalence breeds: American Staffordshire terrier (STA), Bloodhound (BL), Bullmastiff (BMF) Newfoundland (NF), and the Rottweiler (RO) (B) presents the same for the low prevalence breeds German shorthaired pointer (GSP), Flat coated retriever (FR), Belgian tervuren (BT), Belgian sheepdog (BSD) and the Shetland sheepdog (SS).

We assessed the trend of EBV over time to provide a sense of improvement in the hip phenotype and genetic progress. The genetic and phenotypic trend in hip ratings among the high prevalence breeds are presented in Fig 1(A) and that of the low prevalence breeds in Fig 1(B). Note that the right panel of phenotypic change presents the percentage of dogs, by year of birth, scored on hips as excellent. The left panels in the figure, the genetic change in each population, is the average of the estimated breeding values in hip disease for all dogs born in that calendar year. It is important to remember, that the breeding values themselves do not have a direct interpretation to severity of disease. That is because the observation of disease (i.e., the phenotype) is a category, and our analysis links that category on a probit (i.e., normal) scale. Dogs with larger breeding values are, of course, more likely to produce progeny with poorer ratings (that is, higher hip score categories) than dogs with a smaller breeding value. A population that is improving the soundness of hips over time is one with a negative slope across birth years. However, there is no one-to-one correspondence of hip breeding value and a specific rating category for the offspring. Rather, a smaller breeding value translates to an increased probability that any offspring would fall in the categories of “excellent” and “good”. Within high and low prevalence breeds, over the period of evaluation, a reduction in the mean EBV was observed. For the Newfoundland and Rottweiler, this corresponded with a trend to increased numbers of hips rated excellent. In contrast, the Bloodhound, Bullmastiff, and American Staffordshire terrier, while demonstrating a trend of reduced EBVs, have shown little overall change in the percentage of hips rated excellent indicating that the change is likely seen in the other non-dysplastic categories.

Fig 1(B) illustrates a similar trend of improvement in breeds with a low breed prevalence of CHD and therefore characterized for being at low risk for hip disease. As seen in the lower panel of phenotypic trend, as expected, a vast majority of dogs in those breeds were scored as “excellent”. Nevertheless, there was continued genetic improvement in these breeds as reflected in the trajectory of the average EBVs for CHD, most noticeably observed in the Flat coated retriever and the German shorthaired pointer breeds. As in Fig 1(A), the relatively small number of hip score phenotypes available from breeds added a level of variability to the response not seen in the larger population sizes. Yet the trend was consistent, reflecting that although these breeds are not at serious risk of hip disease, breeders remain mindful of this disorder and consider this information in formulating their breeding decisions.

**Fig 2:**
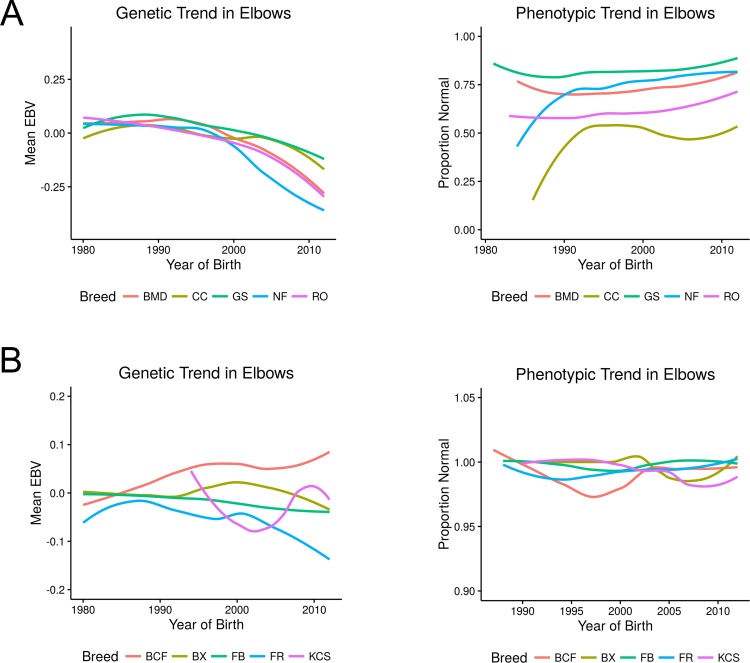
Genetic and phenotypic trends in ED. (A) presents the EBV and the proportion of elbows rated as normal among the high prevalence breeds: Bernese mountain dog (BMD), Chow chow (CC), German shepherd dog (GS), Newfoundland (NF), and the Rottweiler (RO). (B) presents the same for the low prevalence breeds Bichon frise (BCF), Boxer (BX), Briard (FB), Cavalier King Charles spaniel (KCS), and Flat coated retriever (FR).

Fig 2 provides a visualization of the change in ED across five breeds identified as having high prevalence and five breeds having low prevalence. The five breeds with the highest ED prevalence were Chow chow, Rottweiler, Bernese mountain dog, Newfoundland, and German shepherd dog (Fig 2(A)). Note that Chinese shar-pei would also be considered a high-risk breed by the measure of prevalence in this database, however the relative small number of elbow disease phenotypes rendered the ensuing plots erratic and of limited interpretive value. The five breeds considered to be at low risk for ED are Boxer, Flat coated retriever, Bichon, Cavalier King Charles spaniel and Briard. Note, once again, phenotypic change is the percentage of dogs, by year of birth, having normal elbow scores and representing genetic change in each population is the average of the EBVs for ED for all dogs born in that calendar year. As with the EBVs for CHD, breeding values themselves do not have a direct interpretation to severity of disease although dogs with larger breeding values are more likely to produce progeny having higher elbow score categories than dogs with a smaller breeding value. Moreover, a population that is improving the genetic merit for soundness of elbows over time is one displaying a negative slope across birth years. But again, there is no one-to-one correspondence of elbow breeding value and a specific category for the offspring.

Fig 2(B), highlighting the phenotypic and genetic change in those five breeds with the lowest risk of elbow disease, tells a much different story about this disorder and the role selection plays in this disease. Most notable is the proportion of dogs in these breeds scored as “normal”, a proportion hovering near 100% even before the year 2000 and minimal changes in estimated breeding values.

**Fig 3:**
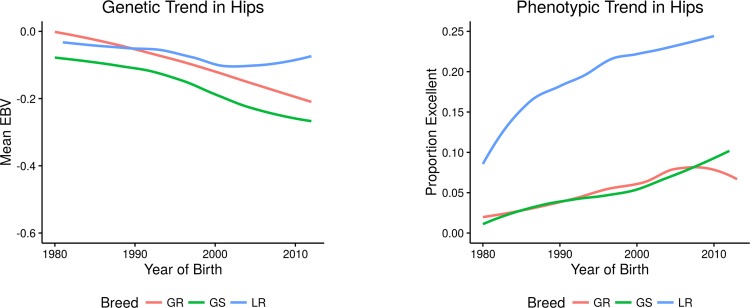
Genetic (EBV) and phenotypic (proportion of hips rated as excellent) trends in CHD for the three breeds with the greatest number of hip evaluations: German shepherd dog (GS, n = 107,048), Golden retriever (GR, n = 133,920), and Labrador retriever (LR, n = 234,382).

To ensure that the findings observed for hip conformation were applicable broadly, breeds having the greatest number of hips assessed (S1 Table) that is, the Labrador retriever, the Golden retriever, and the German shepherd dog, were also analyzed in depth. The hip genetic trend for those three breeds with large population size did not deviate from the trends seen in the breeds reported above indicating that slow, substantive progress has been achieved in hip improvement with incremental changes in elbow conformation (Fig 3).

**Fig 4:**
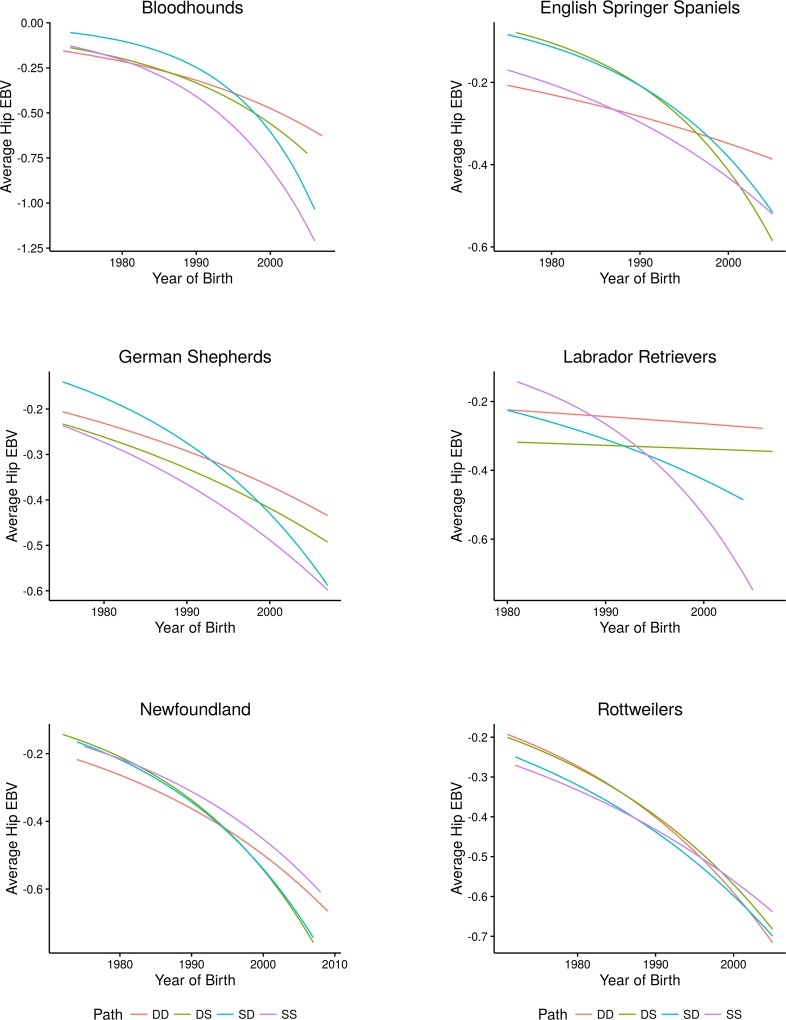
Four path plot representing CHD response to selection through the selection of sires of sires (SS—purple lines), sires of dams (SD—blue lines), dams of sires (DS—olive lines) and dams of dams (DD—orange lines) for breeds (listed alphabetically) representing high prevalence and low heritability (Bloodhound, Newfoundland, and Rottweiler), high representation in the data and above average heritability (German shepherd dogs and Labrador retrievers), and average prevalence and high heritability (English springer spaniel).

The selection patterns derived from evaluating the impact of maternal or paternal selection strategies on progress in CHD are shown in Fig 4. For this analysis to evaluate the impact of the sires of sires (SS), sires of dams (SD), dams of sires (DS), and dams of dams (DD), the Bloodhound, Newfoundland, and Rottweiler were chosen for having high CHD prevalence and below average heritabilities, German shepherd dogs and Labrador retrievers were chosen due to their high representation in the database as well as having above average heritability whereas the English springer spaniel was chosen for having prevalence near the average of the breeds under study and high heritability and submissions sufficient for reliable analyses. German shepherds had. For breeds with low heritabilities applying selection on both sexes improved the population EBV. However, overall, sires proved a greater contributor to improving hip conformation than dams (p < 0.05).

## Discussion

Minimizing the incidence of debilitating orthopedic disorders is paramount, yet past studies that have evaluated CHD and ED heritability report highly variable estimates and nominal response to selection. Contributing factors to disparate heritability estimates include sample size, breed composition, and relatedness of the population studied. The time frame under study, if the evaluation scheme is voluntary or mandatory, and if all evaluations are available for analyses also influence the accuracy of the estimate and assessment of joint improvement. In the absence of DNA-based genetic testing, utilization of estimated breeding values based upon phenotypic radiographic assessment related to CHD and ED has been promoted. Therefore, it is important to best characterize the potential improvement that can be achieved based upon phenotypic selection. The present study was undertaken to comprehensively assess the impact of phenotypic selection on hips and elbows over several decades in multiple dog breeds.

In this study we determined the impact of age and sex on the expression of CHD and ED. Similar to other studies that evaluated fewer breeds and targeted individual dogs [45, 46], increasing age had a subtle negative impact on the ratings for either hips and elbows in a breed specific manner. A previous study of both pure and mixed breed dogs found no difference in CHD between the sexes [5] but interestingly, in the present study, males were more likely to earn a favorable rating than females in breeds with a high prevalence of CHD, a finding also reported by Wood et al in which CHD was more prevalent in Newfoundland but not Flat coated retriever females [47]. It has been suggested that females in estrus have greater joint laxity that may influence the ratings but a prospective study done Keller et al., found that while the Norberg angle measurements showed slightly greater subluxation, the overall joint evaluation rating was unchanged [48]. One plausible explanation for the current observation is that for some breeds females receiving a less favorable rating might be due to the age at which females are neutered. A greater incidence of CHD has been associated with females neutered early in life [49–51]. Additional investigation into a breed-specific sex effect is warranted.

When one considers the phenotypic trend of CHD across the period of available data, all breeds appear to be making progress, which is reflected in the increasing proportion of dogs scored as “excellent”. Not surprisingly the response in the breeds with greater numbers of data points (i.e., Newfoundland and Rottweiler) demonstrate a more stable and consistent increase in this proportion. Breeds with a smaller population size (i.e., American Staffordshire terrier and Bloodhound) display a trend of improvement, but with more instability across years and even a loss of gain in excellent hip phenotypes with the Bloodhound. This instability was also reflected in the changes in average breeding value across time.

As expected, the consistent trend in all five high prevalence breeds, and those breeds with high submissions, showed a decline in the average EBV, remembering that on the scale used here, higher hip scores reflect increasing severity of disease and lower EBVs indicate a trend to improved hip scores. Owing largely to the number of dogs available in each birth year, Newfoundland and Rottweiler demonstrate a steady decline in average breeding value. For breeds with low CHD prevalence, the phenotypic advancement over time was greater than in the breeds with higher prevalence. This evidence suggests that breeders appear to be using the information collected in the various health databases, including health registries managed by specific breed clubs, for making informed choices on breeding alternatives.

Interestingly, the Labrador retriever showed a slower rate of decline in average CHD EBV over the time period studied than that observed in other popular breeds. Yet, at the same time, the proportion of excellent-scored hips increased substantially. We believe that what is demonstrated in this seemingly inconsistent observation is a combination of selection by breeders (i.e. selection against hip disease) and a “migration” of new animals to register with the OFA database (i.e., an influx of animals that may have few relatives established in the database). EBVs are computed with a reference to a base population, that is, a group of animals that do not have parents or the parental phenotype identified. Including new animals without parents or ancestors in the database will necessarily re-orient the base population, impacting the average breeding values over the course of time and altering the scale of genetic progress. Nevertheless, these new “migrants” will contribute their observed phenotypic class to the fraction of animals scored as “excellent”. Of course this combination of selection progress and the inclusion of new animals into the database occurs in all populations, not only Labrador retrievers. Nevertheless, the impact of including new animals in the database with no known ancestors in the pedigree is likely exacerbated in breeds with large populations than one would expect in small population breeds where the natural accumulation of inbreeding will reflect more relationships among the animals in the database.

Taking a broad view of the estimates of heritability for CHD, one might be struck by the relatively high values for this parameter across this sample of breeds, in contrast to other published estimates of this parameter in earlier investigations. The overall range of CHD heritability estimated in the present study was 0.46 to 0.76, somewhat higher than 0.35 and 0.44 reported for Labrador retrievers [52, 53] and much higher than 0.30 for Australian German shepherd dogs [54] and the 0.22 representing a composite estimate of 74 breeds [1]. Yet, the estimates were comparable to the 0.4 to 0.75 reported in other studies [22, 47, 55]. Breed specific heritabilities should not be unexpected given that different breeds present different ranges of physical hip joint measurements associated with the dysplastic condition [56]. Differences in heritability estimates may also reflect that not all previous studies computed heritabilities using a threshold model that was used in the present study.

One explanation for the observed heritability estimate differences may reflect individual evaluation of each breed as opposed to a composite assessment. The overall range of ED heritability similarly reflected breed differences with a high of 0.89 to a low of 0.01. Notably, breeds with low estimates did not reflect prevalence of ED nor limited numbers of dogs assessed. A similar range of ED estimates has also been reported in the literature for multiple dog breeds [30, 32, 57]

Another likely factor contributing to relatively high heritabilities is the sample of animals considered in the analysis. Dogs with data in either of these joint disorder data sets are unlikely to be a random sample of their respective breeds. Indeed, one might reasonably suspect that this sample is more likely to reflect owners interested in the impact of inheritance on these disorders and leveraging that in their breeding decisions. Of course, the method of analysis can also be added as a possible explanation for our relatively high parameter estimates. As outlined in the section on methodology, our analysis reflects the true categorical nature of the OFA scoring system, in contrast with the work of other investigators (e.g., [1]) who evaluated this ordered set of categories as following a multivariate normal likelihood.

Contrasting the heritability values for ED with those for CHD, the difference in range of these values is apparent (0.01 to 0.89 for elbows and 0.46 to 0.76 for hips). The most immediate cause is likely statistical, reflecting the greater number of records available on hip disease than that available for elbow disease. Sample size, compounded by the nature of those owners/breeders who actively participate in contributing records to this database, can have a profound impact on the estimation of variability. In addition, issues of sample size are also reflected in the variability of the ED phenotype itself, where some breeds have a considerable proportion of animals scored as “normal”. Finally, it is important to note, when estimating these genetic parameters in populations spanning several generations, that the estimates also take into account the level of genetic variation in the initial base population (that set of animals in the pedigree without identified parents) rather than directly reflecting the present breeding population [38]. This in turn is influenced by those individuals assessed in the base population, which are generally owned by “first-adopters” of new data resources and interested in reducing inherited conditions.

The findings of the present study demonstrate the variability of breeds and underscore the clear need to consider the individual breed when assessing disease liability. The heritabilities were also analyzed by haplotype relationship [58] to detect trends based upon ancestry but none were apparent (data not shown). Variability in genetic contribution by breed may be a consequence of particular breeding strategies utilized and/or the diversity of the genetic pool of a particular breed. Each breed appears idiosyncratic for the genetic basis of its orthopedic disease. Given the polygenic basis of these disorders, it is not unreasonable to suggest that each breed has its own private set of causative mutations. This supposition is supported by the extensive number of chromosomes showing association with CHD that vary by breeds analyzed; for instance in Portuguese water dogs linkage was identified for CFA 1 and 3 [59, 60], in Labrador retrievers linkage on CFA 1, 2, 5, 8, 10, 15, 20, 21, 22, 25, and 32 [61–63], and CFA 3, 9, 19, 24, 26, 33, and 34 in German shepherd dogs [64, 65]. An alternative explanation may be that common morphology plays a role but the variable morphology seen in the breeds having high or low heritability for either CHD or ED does not support that view.

Genetic correlation is typically interpreted as a measure of pleiotropy in the loci impacting traits as well as genes that may be in linkage disequilibrium with the pleiotropic genes. Therefore, a positive genetic correlation between hip and elbow scores would be the ideal outcome for breeders, implying that as selection pressure is placed on one trait, the other will move in the same direction. Negative correlations impose a more challenging setting for the improvement of both traits simultaneously. The magnitude and direction of a genetic correlation is a reflection not just of the loci involved but also of the allele frequencies that underlie those genotypes. The variability in correlation between CHD and ED reported in the literature and identified here indicates the uniqueness to the breeds with respect to orthopedic conditions. Correlations between the two joints range from 0 to 0.32 [32, 57, 66] to a high of nearly 1. In the absence of an obvious universal association of a correlation value with breed size, popularity, and participation in screening one must conclude the breeds themselves vary in genetic control. This finding again emphasizes the need for breed specific selection programs in order to obtain substantive improvement and also suggests an interplay between the loci that govern a breed’s morphology and these orthopedic conditions that is likely breed specific.

Perhaps the most notable element of the phenotypic trends in ED displayed by the data is, with the exception of the Chow chow breed, the consistent proportion of dogs scored as normal, even within the breeds having a high prevalence of ED. Nevertheless, there was a steady, if small, decline in the average breeding value for ED, appearing to begin with those dogs born near the year 2000. This slight improvement in the genetic merit towards improved elbow conformation may have been associated with the selection pressure placed on hips; this being a correlated response in elbow health mediated by the positive genetic correlation between hips and elbows in these five breeds (refer to Table 3). Among breeds least likely to present with ED, the minimal changes observed in the phenotypic trend was more likely due to the challenges of sampling in populations of small size rather than some unconscious changed facilitated by breeders. Similarly, the change in average breeding values across time demonstrate little, if any, selection pressure on this trait in these breeds thought to be at low risk for elbow disease.

The breeds having a high prevalence CHD all had statistically equivalent heritability estimates for CHD, yet displayed differential reductions in EBVs over time. Similarly, the low prevalence CHD breeds had variable heritability estimates ranging from 0.49 to 0.61 without demonstrating a relationship between improvements in EBVs that might be ascribed to heritability. The same lack of direct relationship of heritability estimate and EBV response was noted for ED. Although one might expect that the breeds with the largest heritabilities would exhibit the greatest response, intensity of selection, population size, use of a popular sire, and selective owner participation in a non-compulsory evaluation scheme all play a role in the EBV calculation.

As with any study, there are limitations to the present analyses. The data reflect only those dogs whose breeders and owners submitted radiographs for analysis by the OFA. An ideal data set would include hip and elbow evaluations for parents and all offspring for all the breeds. The representation of the present population varied by breed; for some breeds the submission rate represented a much higher proportion of the purebred population than for others. A study by Paster et al. [67] suggested that owners were more likely to submit for evaluation radiographs in which the hips appeared normal and that Rottweiler owners were more likely than Golden retriever owners to submit their radiographs. The voluntary database analyzed in this study therefore only included information on dogs that were submitted; dogs that were never evaluated or those that were evaluated but for whom owners elected not to submit were not be captured. Despite efforts by breed clubs and registry bodies to encourage more widespread use of radiographic screening, such as the implementation of the Breeder of Merit or Bred with Heart programs sponsored by the American Kennel Club, without mandatory participation, the data available in the OFA database are incomplete. These particular limitations underscore the need to assess data in a breed specific manner and increase overall participation in the screening scheme. Additionally, Smith et al. [68] identified body weight as a risk factor for CHD in the Labrador retriever, German shepherd dog, Golden retriever, and Rottweiler. Those were the breeds contributing the largest sample size to the global dataset and body weight was not recorded thereby preventing it from being incorporated into the model. Despite that limitation, the findings for those four breeds showed phenotypic improvement over the period of study and mirrored that of the other breeds for which weight may be less of a factor indicating the robust value of the findings.

Globally, CHD is a recognized welfare concern and efforts have been in place for several decades to reduce the prevalence. Ginja et al., 2010 recommend implementing concerted EBV breeding schemes to reduce incidence; these authors also stated that individual dog phenotypic selection has been ineffective in producing change [10]. Although some reviews indicate that the prevalence of CHD has increased [10, 14, 37], that disagrees with other reports that indicate moderate improvement by incorporating phenotypic selection schemes [1, 17, 18, 53, 57]. Genevois et al., report that a reduction of CHD prevalence has been achieved in some, though not all, of the 31 breeds they investigated [3]. A more rapid rate of improvement is clearly desired and in an era of genomic interrogation, reduction in the prevalence based upon mutation based genetic selection tools is on the horizon. However, in the absence of those tools, classical selection focused upon phenotypic characteristics has yielded variable results as reported in the literature. The present study, that indicated a steady improvement in hip and elbow conformation, reinforces the concept that phenotypic selection for hip conformation can be very effective, especially for breeds wherein the voluntary participation in screening efforts is widely embraced (evidenced by, for example, the Labrador retriever, German shepherd dog, Golden retriever, and Rottweiler). Across all breeds, the data presented here indicates a steady improvement in hip and elbow conformation.

Many investigators have advocated the implementation of estimated breeding values (EBV) to accelerate progress in the selection process [1, 14–16, 19, 53, 54, 69–71] suggesting that genomic selection would improve the response to selection up to three times more rapidly than phenotypic selection [72]. The inherent differences of response for the different breeds, would argue that the EBV breed specific approach is necessary. The data also suggest that any genetic-based test development will need to be breed specific. Any genetic-based test development for complex traits, in addition to being breed specific, would benefit from comprehensive phenotyping of the population [73] including body weight, growth rate, and neuter status.

The present data indicate the effectiveness of concerted selection schemes based upon phenotypic assessment in improving joint health and would suggest emphasizing selection on sires, a practice currently employed by breeders. One may argue that sires produce more offspring and may skew the interpretation. However, importantly, the four path plot analyses that demonstrate the role of sire selection are not weighted by the number of grand-progeny a given dog has in the population. Thus, the data suggest that more rapid improvement may be achieved by emphasizing the health characteristics of the sires. One must be mindful of avoiding a popular sire effect. Popular sires are the greatest contributor to the spread of genetic disorders [74] yet prudent sire selection appears to exact the most rapid improvement for the complex condition of CHD.

The data presented here confirms that employing phenotypic health information and selecting sires and dams from pedigrees free from dysplasia does reduce the condition. Acceptance of using health information in breeding decisions is growing. A 2004 study of Dutch Boxer breeders [75] indicated that 32% of the breeders utilized genetic information, expressed as an odds ratio of particular sire-dam combinations producing deleterious health traits, in their mate selections. A recent report assessing selection practices among Australian dog breeders indicated that the “genetics and health” attribute of potential dams was one of the top four decision components [76]; despite this weighting in dam selection some breeders failed to practice health screening, including for CHD. As selection tools for health characteristics improve, using those will demonstrably improve the health of dogs.

## Supporting information

S1 Table Descriptive statistics of the population under study. (PDF)

S2 Table Impact of sex and age on the CHD and ED scores. (PDF)

S3 Table Data for all breeds by year. (XLSX)
