# Supplementary material for: Long-term genetic selection reduced prevalence of hip and elbow dysplasia in 60 dog breeds
Source: PLoS One. 2017 Feb 24;12(2):e0172918. doi: 10.1371/journal.pone.0172918 (PMC5325577; doi:10.1371/journal.pone.0172918)
Supplement: S1 Table — (PDF) [file pone.0172918.s001.pdf]

S1 Table. Descriptive statistics of the population under study.

Breed	Hips (n)	% normal hips	%CHD	Elbows (n)	% normal elbows	% ED
Akita	16142	87.74	12.26	2311	98.87	1.13
Alaskan malamute	13942	89.17	10.83	909	97.03	2.97
American Staffordshire terrier	2952	75.61	24.39	753	83.93	16.07
Anatolian	1924	90.23	9.77	575	94.78	5.22
Australian cattle dog	3631	86.56	13.44	1224	90.20	9.80
Australian shepherd	33353	94.67	5.33	7778	96.50	3.50
Bearded Collie	4510	94.70	5.30	670	98.06	1.94
Belgian Malinois	2890	94.84	5.16	1749	91.37	8.63
Belgian sheepdog	4152	97.18	2.82	1924	95.48	4.52
Belgian tervuren	6072	96.92	3.08	3416	96.11	3.89
Bernese mountain dog	18516	84.98	15.02	13453	74.04	25.96
Bichon	3606	94.12	5.88	664	99.55	0.45
Bloodhound	2830	75.34	24.66	1173	86.87	13.13
Border collie	11819	89.90	10.10	2834	98.87	1.13
Bouvier	8134	85.87	14.13	3246	92.17	7.83
Boxer	5540	89.30	10.70	686	99.27	0.73
Briard	2456	86.97	13.03	627	99.84	0.16
Brittany	18532	86.26	13.74	1212	98.35	1.65
Bullmastiff	5560	76.03	23.97	2415	85.80	14.20
Cavalier King Charles spaniel	6662	88.65	11.35	599	99.67	0.33
Chesapeake bay retriever	12882	80.57	19.43	2594	94.76	5.24
Chinese shar-pei	9571	86.90	13.10	597	76.05	23.95
Chowchow	5296	80.83	19.17	987	51.37	48.63
Doberman pinscher	15882	94.33	5.67	2428	99.18	0.82
English setter	10503	84.94	15.06	2940	85.78	14.22
English Springer	15165	87.78	12.22	2520	87.46	12.54
Flat coated retriever	5698	96.26	3.74	2500	99.32	0.68
German Shepherd	107048	81.06	18.94	37233	82.17	17.83
German shorthaired pointer	16310	96.13	3.87	2186	99.22	0.78
German wirehaired pointer	4165	91.98	8.02	739	97.83	2.17
Giant schnauzer	4350	83.38	16.62	546	91.58	8.42
Golden retriever	133920	81.16	18.84	35401	90.31	9.69
Gordon setter	6094	81.72	18.28	907	87.43	12.57
great dane	13004	88.38	11.62	2464	96.47	3.53
Great Pyrenees	6008	91.29	8.71	896	98.55	1.45
Greater Swiss mountain dog	2776	84.62	15.38	2350	90.89	9.11
Havanese	3518	93.75	6.25	1961	94.54	5.46
Irish setter	11352	88.82	11.18	648	97.22	2.78

Irish water spaniel	1369	89.04	10.96	545	84.40	15.60
Irish wolfhound	1903	95.53	4.47	756	87.70	12.30
Keeshond	4816	94.25	5.75	1113	92.63	7.37
Labrador retriever	234382	88.76	11.24	72126	90.43	9.57
Leonberger	1793	87.28	12.72	1567	96.75	3.25
Mastiff	10794	81.44	18.56	6114	86.87	13.13
Miniature American Australian shepherd	1789	93.40	6.60	641	97.82	2.18
Newfoundland	15359	75.25	24.75	6682	77.30	22.70
Nova Scotia Duck Tolling Retriever	2084	94.19	5.81	796	97.49	2.51
Old English sheepdog	10712	82.24	17.76	506	96.64	3.36
Pembroke Welsh corgi	11083	83.44	16.56	789	97.08	2.92
Poodle	24039	89.01	10.99	2386	97.19	2.81
Portugese water dog	8132	89.02	10.98	3008	98.57	1.43
Rhodesian Ridgeback	11641	95.58	4.42	5979	95.05	4.95
Rottweiler	92718	79.93	20.07	16162	61.93	38.07
Samoyed	16131	89.71	10.29	1463	98.15	1.85
Shetland sheepdog	20668	95.84	4.16	825	97.21	2.79
Spinone Italiano	1234	86.22	13.78	565	94.34	5.66
Tibetan mastiff	989	85.74	14.26	513	86.16	13.84
Viszla	14191	93.81	6.19	2063	98.01	1.99
Weimaraner	12158	92.06	7.94	1652	98.43	1.57
Welsh springer spaniel	2102	89.20	10.80	763	98.56	1.44
