# Supplementary material for: Long-term genetic selection reduced prevalence of hip and elbow dysplasia in 60 dog breeds
Source: PLoS One. 2017 Feb 24;12(2):e0172918. doi: 10.1371/journal.pone.0172918 (PMC5325577; doi:10.1371/journal.pone.0172918)
Supplement: S2 Table — (PDF) [file pone.0172918.s002.pdf]

S2 Table. Impact of sex and age on the CHD and ED scores.

Breed	Sex Impact on Hip Score[1]	Sex Impact on Elbow Score[1]	Age Impact on Hip Score[2]
Akita	-0.039 ± 0.03	**0.728 ± 0.282**	0.005 ± 0.001
Alaskan malamute	**-0.342 ± 0.036**	**1.013 ± 0.267**	0.002 ± 0.001
American Staffordshire terrier	**0.172 ± 0.07**	-0.195 ± 0.16	**0.005 ± 0.002**
Anatolian	-0.014 ± 0.092	0.003 ± 0.282	**0.011 ± 0.004**
Australian cattle dog	0.116 ± 0.068	0.009 ± 0.167	**0.007 ± 0.002**
Australian shepherd	**0.046 ± 0.023**	**0.176 ± 0.08**	**0.002 ± 0.001**
Bearded Collie	**0.375 ± 0.06**	0.487 ± 0.338	**0.009 ± 0.002**
Belgian Malinois	-0.003 ± 0.082	0.225 ± 0.13	0 ± 0.003
Belgian sheepdog	**0.15 ± 0.062**	**0.404 ± 0.15**	0.003 ± 0.002
Belgian tervuren	0.077 ± 0.051	0.216 ± 0.113	0.002 ± 0.002
Bernese mountain dog	-0.011 ± 0.027	0.031 ± 0.035	**0.016 ± 0.002**
Bichon	**-0.225 ± 0.068**	-0.27 ± 0.698	**0.005 ± 0.002**
Bloodhound	**-0.232 ± 0.073**	-0.013 ± 0.136	0.002 ± 0.003
Border collie	**0.140 ± 0.038**	**0.501 ± 0.214**	**0.006 ± 0.001**
Bouvier	**-0.098 ± 0.041**	-0.05 ± 0.106	**0.008 ± 0.002**
Boxer	0.002 ± 0.066	-0.315 ± 0.54	0.001 ± 0.003
Briard	0.125 ± 0.076	-10.842 ± 5.958	**0.007 ± 0.003**
Brittany	-0.057 ± 0.030	-0.099 ± 0.284	**0.005 ± 0.001**
Bullmastiff	**-0.108 ± 0.050**	**0.299 ± 0.105**	**0.005 ± 0.002**
Cavalier King Charles spaniel	**0.211 ± 0.052**	-0.665 ± 0.555	**0.005 ± 0.002**
Chesapeake bay retriever	-0.034 ± 0.035	**0.365 ± 0.117**	**0.006 ± 0.001**
Chinese shar-pei	**-0.129 ± 0.045**	0.221 ± 0.175	**0.011 ± 0.002**
Chowchow	**0.119 ± 0.054**	0.114 ± 0.107	**0.005 ± 0.002**
Doberman pinscher	**0.077 ± 0.033**	0.202 ± 0.254	0 ± 0.001
English setter	**0.109 ± 0.037**	**0.468 ± 0.094**	0 ± 0.001
English Springer	**-0.003 ± 0.035**	**0.469 ± 0.112**	0.002 ± 0.001
Flat coated retriever	-0.027 ± 0.051	0.539 ± 0.372	**0.005 ± 0.002**
German Shepherd	-0.141 ± 0.034	**0.364 ± 0.051**	0.003 ± 0.001
German shorthaired pointer	**0.101 ± 0.032**	0.374 ± 0.259	0.002 ± 0.001
German wirehaired pointer	0.026 ± 0.06	0.049 ± 0.306	0.004 ± 0.002
Giant schnauzer	**-0.299 ± 0.059**	**0.674 ± 0.222**	**0.007 ± 0.003**
Golden retriever	-0.002 ± 0.036	**0.21 ± 0.075**	**0.01 ± 0.001**
Gordon setter	**0.215 ± 0.051**	**0.412 ± 0.149**	**0.01 ± 0.002**
Great dane	-0.003 ± 0.033	-0.151 ± 0.148	0.002 ± 0.002
Great Pyrenees	-0.025 ± 0.051	0.455 ± 0.362	0.002 ± 0.002
Greater Swiss mountain dog	0.132 ± 0.072	-0.235 ± 0.126	**0.018 ± 0.005**
Havanese	-0.02 ± 0.074	0.246 ± 0.246	0.004 ± 0.003
Irish setter	**0.183 ± 0.035**	0.543 ± 0.537	**0.006 ± 0.001**

Irish water spaniel	0.118 ± 0.109	0.265 ± 0.19	0.005 ± 0.005
Irish wolfhound	-0.156 ± 0.099	**0.457 ± 0.221**	-0.002 ± 0.004
Keeshond	0.037 ± 0.06	0.186 ± 0.158	**0.007 ± 0.002**
Labrador retriever	-0.013 ± 0.034	**0.241 ± 0.063**	0.002 ± 0.001
Leonberger	-0.176 ± 0.107	-0.112 ± 0.183	**0.026 ± 0.006**
Mastiff	-0.016 ± 0.037	0.074 ± 0.063	0.009 ± 0.002
Miniature American Australian shepherd	-0.024 ± 0.096	0.147 ± 0.408	0.003 ± 0.004
Newfoundland	**-0.058 ± 0.029**	**0.203 ± 0.055**	**0.009 ± 0.001**
Nova Scotia Duck Tolling Retriever	**-0.197 ± 0.1**	0.196 ± 0.297	**0.014 ± 0.004**
Old English sheepdog	-0.029 ± 0.04	0.361 ± 0.284	**0.005 ± 0.002**
Pembroke Welsh corgi	**-0.221 ± 0.037**	0.118 ± 0.269	**0.006 ± 0.001**
Poodle	0.029 ± 0.027	0.249 ± 0.187	**0.006 ± 0.001**
Portugese water dog	**0.099 ± 0.041**	0.168 ± 0.167	**0.014 ± 0.002**
Rhodesian Ridgeback	**0.175 ± 0.037**	**-0.246 ± 0.085**	**0.005 ± 0.002**
Rottweiler	**-0.163 ± 0.029**	**0.39 ± 0.047**	**0.003 ± 0.001**
Samoyed	-0.037 ± 0.029	-0.267 ± 0.247	**0.005 ± 0.001**
Shetland sheepdog	**-0.207 ± 0.031**	0.066 ± 0.275	**0.007 ± 0.001**
Spinone Italiano	**-0.337 ± 0.112**	-0.03 ± 0.257	0.005 ± 0.005
Tibetan mastiff	0.076 ± 0.145	**0.402 ± 0.194**	-0.006 ± 0.005
Viszla	**0.215 ± 0.037**	0.068 ± 0.231	-0.001 ± 0.001
Weimaraner	**-0.146 ± 0.035**	0.294 ± 0.247	0.002 ± 0.001
Welsh springer spaniel	-0.007 ± 0.089	0.884 ± 0.745	0.001 ± 0.004

[1]Bold numbers indicate significantly different from zero. Positive values reflect a decreased risk of CHD or ED for females and negative values reflect a decreased risk in males

[2]Bold numbers indicate significantly different from zero and positive values reflect an increased risk of CHD or ED for older animals and negative values reflect an increased risk in younger animals
